# The Relations of Physicians to Their Medical Supplies

**Published:** 1887-03

**Authors:** 


### THE RELATIONS OF PHYSICIANS TO THEIR MEDICAL SUPPLIES.

"There are a large number of physicians who, for both therapeutics and materia medica, depend largely—if not mainly—upon the traveling salesmen and their pamphlets and lists, and on the advertising pages of the medical journals. The relations of this class to their supplies is most simple and most favorable. They come in very direct and very close contact with the sources of their supplies, and have much less trouble than any other class of artisans. With others smarter, more ingenious and more plausible than they, to think for them, and then to apply vigorous mercantile principles to their wants thus suggested for them, they have the least practicable amount of thinking for themselves to do, in regard to their remedies and the novelties of the day, and therefore, as they argue, more time to think of and study out their cases. To this class the ready made prescriptions in the form of beautifully colored and coated pills, or palatable solutions and mixtures, do not appeal simply as gratifying various degrees of laziness, or indisposition to think for themselves, but they present themselves as true labor-saving devices, skilfully prepared for the over-worked ability to use them, and as giving more time for the higher and more scientific reaches of the profession." * * * *
—*The Ephemeris.*

A gentleman who, like Dr. Squibb, has received so much gratuitous advertising from the medical press should be the last to attempt to undermine the prosperity of Medical Journals.

The above is evidently written in spite. Others have succeeded where Dr. Squibb failed, and such a wail, little becomes him. It is an insult to the medical profession; its tendency is obviously to ridicule physicians who profit by the skill of the pharmacist who put into convenient and elegant form for administration, many of the combinations and formula found by experience of able physicians to be valuable. Dr. Squibb, would seemingly advocate the old stage-coach days of "every Doctor his own compounder," and because too, other pharmacists by energy, skill and the liberal expenditure of money, have secured the patronage of many of the more enlightened part of the profession. We regard the manufacturing pharmacist as a benefactor, a valuable ally to the prescriber, and nothing but envy,or the "garrulousness of old age," it seems to us, could have inspired such an attack on those who *have been* his friends.

Dr. Squibb seeks also to create the impression that the medical journal is not the proper channel for manufacturers of medicines to use for conveying to the profession information regarding their products. Why not? Where else could they be so well brought to the notice of reading and thinking Doctors. In doing this he strikes a blow at the rights of manufacturers, and at a legitimate source of income to medical journals, which we think should not be submitted to without protest. Many manufacturers of medicines are liberal advertisers in medical journals, and we shall always maintain that the journals are the proper medium for communication between the manufacturers and the physician. Dr. Squibb could as consistently condemn the manufacture of any other commodity out of the crude material, and the advertising of it in appropriate channels—as the conversion of crude drugs into elegant coated pills or elexir—ready-made clothing, for instance; a gentleman should not buy a ready-made suit; he should buy the material and make it, or have it made; he should *think out* the best mode. It should not be advertised where wearers of clothing could see it, but should be kept in the store, and the gentlemen should go to look for it. Out on such reasoning; it is the grumbling of a disappointed man who has not kept pace in late years with the times. Wé recall with much distinctness how he analysed *coca* a short time ago, and published that it contained nothing not to be found in tea or coffee; since which time the now famous cocaine has been manufactured by Dr. Squibb by the ton, after some one else had found it.

We trust that the above unjust and ungenerous reflections by Dr. Squibb upon the medical profession, an unwarranted attack upon the medical press and upon their patrons, the manufacturing druggists, will not be allowed to pass unnoticed by the press, but that it may receive the condemnation which it deserves.

